# ‘Dementia has got two faces’: grief as an experience of holding on and letting go for people living with primary progressive aphasia and posterior cortical atrophy

**DOI:** 10.1080/13607863.2025.2502793

**Published:** 2025-05-18

**Authors:** Claire Waddington, Henry Clements, Sebastian Crutch, Martina Davis, Jonathan Glenister, Emma Harding, Erin Hope Thompson, Jill Walton, Joshua Stott

**Affiliations:** a Dementia Research Centre, Institute of Neurology, University College London, London, UK; b Clinical Education and Health Psychology, University College London, London, UK; c Rare Dementia Support, Dementia Research Centre, Institute of Neurology, University College London, London, UK; d The Loss Foundation, UK; e ADAPT Lab, Clinical Education and Health Psychology, University College London, London, UK

**Keywords:** Qualitative, primary progressive aphasia, posterior cortical atrophy, dementia, grief

## Abstract

**Background:**

research on grief in people with primary progressive aphasia (PPA) and posterior cortical atrophy (PCA), is limited, despite the unique challenges these individuals face due to lack of understanding of their condition, younger age at onset and atypical symptom profile. The current study explores the losses people living with PPA or PCA experience and what helps to navigate these losses.

**Methods:**

in-depth semi-structured research conversations were conducted with 14 participants (*n* = 8 PCA, 6 PPA) to explore experiences of grief and loss related to their dementia. Data was analysed using abductive thematic coding techniques.

**Results:**

the impact and navigation of loss is reflected across five interconnecting themes: what I have lost, am losing and will lose, shared and unique sense of loss, balance between what is lost and what remains, changes in relationships and what helps in navigating loss.

**Conclusions:**

the dynamic interplay between what participants had lost and what they held on to carries significant implications for the design and delivery of support. These findings will be used alongside existing grief theory and interventional frameworks to develop a psychosocial intervention for people living with dementia.

## Introduction

Grief includes psychological, behavioural, social, spiritual and physical responses to loss (Harris & Winokuer, 2019; Rando, 2000). Grief in carers of people living with dementia has been highlighted as a topic of importance in the research literature (Crawley et al., 2023), however there is currently a lack of qualitative research on the experience of grief in people living with dementia (Waddington et al., 2024).

A recent review found that people living with dementia might grieve several previous, current and anticipated losses, including loss of independence and their imagined future (Waddington et al., 2024). Grief may also relate to loss of abilities due to symptom progression, confidence and self-esteem, sense of meaning and purpose, and changes in identity, autonomy, roles and relationships (Alzheimer Society of Canada, 2019; Doka, 2010; Górska et al., 2018; Rentz et al., 2005; Robinson et al., 2005).

People living with dementia experience disenfranchised grief (Doka, 2010; Doka & Aber, 1999) as losses are often not acknowledged, validated or understood by others, leading to increased feelings of loneliness and isolation (Alzheimer Society of Canada, 2019; The Irish Hospice Foundation, 2016). They are more likely to be excluded from support systems typically offered to and accessed by those perceived by society as grieving, such as counselling and grief support groups, due to the assumptions that this support will not be relevant or appropriate for individuals living with cognitive impairments (Rentz et al., 2005; Sass et al., 2022; Shoesmith et al., 2022). Given the potential ubiquity and negative impacts of this grieving experience, it is critical to understand this better to improve support. Additionally, while grief in people living with all forms of dementia is poorly understood, the experience of, and strategies for navigating grief in less common, non-memory-led forms of dementia is particularly neglected and will be the focus of the current paper.

Primary progressive aphasia (PPA) is a group of conditions that lead to progressive loss of language abilities, alongside behavioural and cognitive changes (Marshall et al., 2018). PPA is typically classified into three clinical subtypes: non-fluent variant (nvPPA), semantic variant (svPPA) and logopenic variant (lvPPA) (Gorno-Tempini et al., 2011). People living with PPA often develop symptoms under the age of 65, bringing additional challenges related to life stage, employment, finances and comparatively younger families (Mayrhofer et al., 2021; Svanberg et al., 2010; Van Vliet et al., 2013). Memory may be relatively well preserved in the early stages of PPA, alongside insight and awareness of symptoms and progression, which likely contributes to high rates of depressive symptoms (Collins et al., 2023; Marshall et al., 2018; Medina & Weintraub, 2007). Previous research indicates that grief is an important component of the experience of living with PPA (Volkmer et al., 2023), and that people living with PPA may be experiencing multicomponent grief relating to activities, roles and perceived sense of self (Lo et al., 2022). People living with behavioural variant frontotemporal dementia (bvFTD) were not included in the current study, as reduced insight is a commonly reported symptom in this population (Wilson et al., 2016), and awareness of symptoms and diagnosis was an essential component of participation, given the research questions.

Posterior cortical atrophy (PCA) is a condition that initially involves difficulties with visual, perceptual and spatial skills (Benson et al., 1988). PCA often occurs before the age of 65 and is usually caused by an atypical form of Alzheimer’s disease (Chapleau et al., 2024). As with PPA, insight and memory are often relatively preserved in the early stages (Schott et al., 2017; Yong et al., 2023) and depression and anxiety are commonly experienced (Suárez-González et al., 2016), alongside loss of confidence and sense of purpose (Yong et al., 2023). In the early stages, people living with PCA are often aware of their symptoms and the progressive nature of their diagnosis and may also be concerned about the impact on others, with fears of being or becoming ‘a burden’ (Harding et al., 2018).

As PCA and PPA are rarer forms of dementia, there is typically a longer diagnostic process (Marshall et al., 2018; Yong et al., 2023) and increased difficulty accessing appropriate support, as services are primarily tailored to people over 65 with memory issues. While people living with all forms of dementia are likely to experience disenfranchised grief (Doka, 2010), this is likely more prevalent in people living with PCA or PPA, given the lack of wider understanding and awareness of these conditions. This study aims to address this research gap and build on previous findings on the experience of grief in people living with dementia (Waddington et al., 2024).

### Research questions

1. How do people living with early-stage PPA or PCA experience grief and loss related to their diagnosis?
2. What support, strategies and adaptations do people living with these diagnoses find helpful in navigating their experience of grief and loss?

## Materials and methods

Given the exploratory nature of this study and the individual nature of grief, a qualitative design was selected, using semi-structured interviews. People living with dementia were involved in all stages of study development, from conception to publication. The term ‘research conversation’ rather than ‘interview’ was selected following feedback that ‘interviews’ had intimidating connotations. This terminology will be used throughout.

### Participants

Participants had to meet the following criteria:

1. Living with a diagnosis of early-stage PPA or PCA
2. Able to take part in the informed consent process, recorded via video call
3. Able to communicate verbally or via written format (using the chat function in Zoom)

### Recruitment

All participants were recruited *via* the Rare Dementia Support service, which offers specialist social, emotional and practical support services for people affected by a rare dementia diagnosis, both individually and in group settings. The lead researcher attended four (PPA (x2) and PCA (x2)) online peer support group sessions, providing information about the study. Potential participants contacted the researcher directly *via* email and were then provided with further information about the study and consenting procedures.

### Ethical considerations

This study is part of the larger Rare Dementia Support Impact Study, approved by the University College London Research Ethics Committee (8545/004). All participants were provided with an information sheet and given time to read this on their own or together with a research partner before a video-recorded consent call. Capacity to consent was assessed during this videocall, where participants were asked to tell the researcher in their own words what they understood would be involved if they chose to participate. Participants were informed they had the right to withdraw and that their participation or withdrawal did not affect support service provision.

### Procedure

Semi-structured research conversations ranged from 30–75 min (s*ee Appendix* for full research conversation schedule*),* were conducted by the first author (CW) over Zoom, and were held between November 2022 and March 2023. Videocalls were used in accordance with ethical approval guidelines, providing participants with both visual and verbal prompts throughout. Conversations were video-recorded *via* Zoom, transcribed verbatim using the automated transcription process, manually checked and corrected for errors and anonymised.

### Accessibility

Participants were informed they could take breaks or skip questions at any point. The researcher monitored their wellbeing throughout, addressing signs of distress or fatigue. Participants received and responded to questions verbally and/or by typing using the videoconference chat function. Two participants used the chat alongside verbal responses. As verbal communication abilities varied between participants, the level of detail captured within the research conversation data was wide-ranging.

The researcher debriefed with each participant and offered a follow-up support call in case of distress. No participants requested a follow-up call or reported feeling distressed although some reported feeling tired. Several participants reported they found the process therapeutic to be listened to and share their personal experiences.

Due to the sensitive nature of the research conversations, participants were encouraged to speak one-to-one with the researcher if they were comfortable and able to. However, participants were also able to have a research partner present during the research conversation if they preferred. Three participants chose to do so. Any instances of proxy communication *via* the research partner were checked and reflected to the participant to ensure accuracy. This process was noted and addressed separately throughout the analysis.

### Reflexivity

CW conducted all research conversations and the initial transcript analysis. She has prior experience interviewing people living with dementia as part of the broader RDS Impact Study and has over seven years of experience supporting people affected by dementia. To enhance reflexivity, she engaged in ongoing reflexive journaling throughout the research process. Additionally, themes and analyses were discussed iteratively with the research team and further refined through consultations with people living with dementia *via* the Rare Dementia Support Focus Group.

### Data analysis

**Figure 1.**
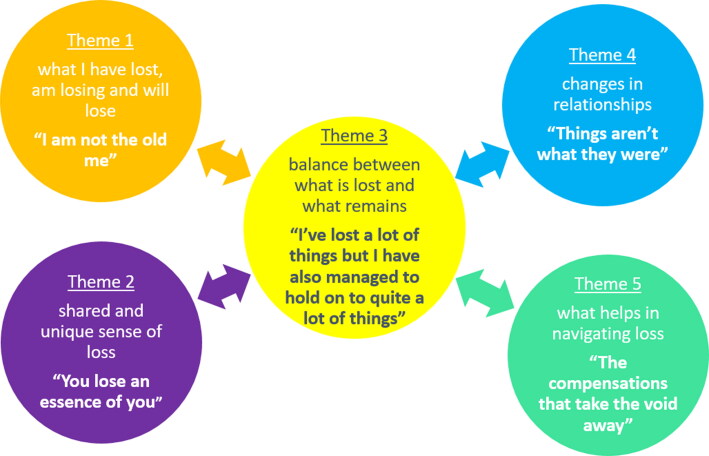
Visualisation of study themes, with the balance between what is lost and what remains being the central and interconnecting theme.

Research conversation transcripts were uploaded to NVivo (v12) and analyzed using both deductive and inductive coding techniques, with metasynthesis findings on grief in dementia (Waddington et al., 2024) forming the deductive thematic grounding. Following reflexive thematic analysis guidelines (Braun & Clarke, 2019), transcripts were read several times, followed by line-by-line coding of each transcript. Individual codes were grouped into broader overarching themes, which were then further refined. These higher-level themes were discussed together with the research team, and with people living with dementia individually, and in a focus group setting. This allowed for further adaptations and to ensure that the terminology was appropriate. The inter-connected nature of the higher-order themes is visualised in Figure 1.

### Sample

Findings represent the perspectives of six participants living with PPA (2 Male, 4 Female) and eight participants living with PCA (4 Male, 4 Female), aged 51–74 (*M* = 63.28, SD = 8.21). Time since diagnosis ranged from five months to five years (*M* = 23 months, SD = 18.58). Most participants were White British (*n* = 12), married (*n* = 11) and heterosexual (*n* = 13). Further details are presented in Table 1.

**Table 1.** Participant characteristics.

Diagnosis	8 posterior cortical atrophy
	6 primary progressive aphasia
Time since diagnosis	5 months − 5 years (*M* = 23 months, SD = 18.58)
	<1 year: 6
	1–2 years: 3
	2–3 years:
	3–4 years: 2
	4–5 years: 2
	> 5 years: 1
Age	Age: 51–74 years (*M* = 63.28, SD = 8.21)
	50–55: 4
	56–60: 2
	61–65: 1
	> 65: 7
Gender	8 Female
	6 Male
Sexuality	13 Heterosexual
	1 Homosexual/Gay/Lesbian
Ethnicity	12 White British
	2 Any Other White Background
Marital status	Married: 11
	Single: 2
	Widowed: 1
Employment	Unemployed: 8
	Volunteering: 3
	Employed: 3

## Results

Results are categorised into five themes (see Figure 1), outlined below and with further illustrative quotes in the *Appendix.*

***Theme 1:*** what I have lost, am losing and will lose: **‘I am not the old me’**

Participants spoke about losses they had experienced before having any symptoms of dementia, their response to receiving a diagnosis of dementia, and fears for future losses.

*Subtheme 1:* losses that came before my diagnosis – ***‘they live with me, even now’***

Several participants shared that previous experiences of loss (e.g. miscarriage, redundancy and bereavement) shaped the way they were navigating current losses, with bereavement specifically contributing to a re-framing of the preciousness of life and end-of-life preferences.

my wife…she got a terminal illness…()…she was an exemplar of how to do it in a way that made people around her feel better….I can only seek to emulate some of the attitude that she had…because I think having a positive look at my prospects is the best way for me to be good to myself…

P3 (PPA)

Two participants shared that they did not feel they had experienced loss before their diagnosis.

*Subtheme 2:* what does my diagnosis mean? – ***‘it’s a terrible shock at the start’***

Most participants felt shocked at receiving a confirmed diagnosis. They contrasted their experience to those living with more typical forms of dementia, and other conditions such as cancer. Several participants felt relieved when diagnosed, as it provided an explanation after a long period of uncertainty. One participant *‘felt free’* (P7 [PCA]) following their diagnosis, as they no longer needed to find and retain employment, which had been challenging.

For several participants, the diagnosis shattered hopes of a treatable cause of their symptoms, making them *‘really start taking these losses into account’* because *‘you can’t make it better’* (P12 [PCA]). Several participants were also initially *‘in denial’* (P10 [PPA]), searching for other causes. Participants felt angry and frustrated at the time it took to get the diagnosis and *‘that nobody seemed able to help’* (P13[PCA]). It took time to process and accept the diagnosis, and to choose who to share this with. Several participants were still navigating this process.

*Subtheme 3:* what does my future hold? ***– ‘redefining who I was going to be’***

Participants spoke about a changed future, a potentially shortened lifespan, and a loss of hope for retirement plans. The diagnosis also triggered fears for the loss of independence, the future burden that this may place on others, and the uncertainties of how and when symptoms might progress.

The unpredictable nature of dementia was a source of distress, with one participant speaking specifically about thoughts related to assisted suicide following their diagnosis. Knowing people with dementia who were at a later stage also led participants to imagine what their future might hold, with a fear that *‘this is going to be me’* (P4 [PCA]).

Several participants chose to try to plan for and control what they could and to focus more on the present as *‘…you just don’t know, do you? So, there’s no point thinking about it too much….because what you expect to happen probably won’t happen and something else will happen…’* (P5 [PCA]).

***Theme 2:*** shared and unique sense of loss **– ‘you lose an essence of you’**

Participants living with PCA shared losses related to getting dressed, household chores, walking independently, and personal care (e.g. cutting nails). Participants living with PPA shared the loss of finding the right words in conversations and participating in social activities independently. Losses were particularly challenging if the activity held personal value and meaning, and when there were no current adaptations to compensate. Losses that may appear small to others had a profound impact on the individual.

…. I no longer could cut the grass….now that seems very sort of juvenile in one sense…but I’ve been cutting the grass since I was 12…you get used to it, because there are obvious reasons why I can’t…but it doesn’t….take away the fact that it isn’t there anymore…

P14 (PCA)

*Subtheme 1:* communication difficulties – ***‘I lost my sense of putting out who I was’***

All participants living with PPA, and several living with PCA, experienced some loss of communication, which was viewed as an important component of previous roles and identity. Difficulties led to a sense of isolation, uselessness and exhaustion. Participants felt a sense of losing the ability to share important aspects of themselves, their personality and their humour with others, which was described as a *‘life sentence’* (P11 [PPA]).

In some cases participants would *‘have to think is it worth the effort’* before trying to communicate, and ‘*felt a feeling of loss that I’d metaphorically lost my voice as well as literally…’* (P3 [PPA]), leading to withdrawal from certain activities that felt too overwhelming or exhausting.

Several strategies were used to assist with communication difficulties, including asking others for assistance. There was a general sense that different strategies might work in different contexts and that what worked for an individual might not work for others.

*Subtheme 2:* loss of employment – ***‘I didn’t have a reason for being’***

Three participants were still employed. Several participants had retired before their diagnosis, and others stopped working due to dementia-related symptoms. Work was a source of pride for most participants. Loss of employment was connected with the importance of leaving a lasting legacy, the loss of the hoped-for future, ‘*loss of …the friendships and the buzz of the work itself’* (P13 [PCA]).

Not all aspects of employment were associated with loss. One employed participant shared that they had received *‘love and support*’ (P1 [PPA]) after sharing their diagnosis with colleagues. Two participants who had stopped working due to their diagnosis shared that it was *‘a loss that I don’t mind losing’* (P7 [PPA]). Another participant also shared that *‘…some friends, I think are meeting up with me a bit more actually….maybe because I’m not working now…’* (P10 [PPA]).

*Subtheme 3:* driving cessation – ***‘it’s a freedom which just disappears’***

Several participants felt that driving cessation was a significant personal loss. This reduced access to community services and was particularly difficult for rural participants. While some participants understood why they could no longer drive, others felt that they had not been able to decide for themselves to stop, and that they still felt *‘safe driving’* (P2 [PPA]). Driving was not only seen as a practical task, but also enjoyable, a social activity and a hobby, and was associated with freedom, spontaneity, independence and connection to others.

However, many participants shared that they were still able to go to the pub, to the gym, and use buses and trains, often with the support of friends and family members. One participant shared that while driving may have led to some sense of loss, *‘…I can cope with that loss…that’s not a difficult one to let go…’* (P5 [PCA]).

***Theme 3:*** balance between what is lost and what remains – **‘I’ve lost a lot of things, but I’ve also managed to hang onto quite a lot of things’**

The third theme is the central and interconnecting theme on the fluctuation between what participants lost and what they held on to. Participants focused on their losses some days more than others, with specific situations being particularly triggering, such as being unable to do everyday tasks. Several participants shared that while they had initially focused more on what they had lost, they now felt able to focus more on the abilities and skills that they had.

*‘…I’m feeling that whilst I’ve lost some things I want to do, I may gain some things because of my new outlook on the world…’*

P3 (PPA)

*Subtheme 1:* challenging own abilities – ***‘don’t look at your mountain, climb it’***

It was often a difficult balance between giving up on activities or persevering, and trying to reduce frustrations while also enabling continued connections with activities, to avoid *‘creating another form of isolation’* (P12 [PCA]).

…I can still read…I can walk, I can run… and…like physically…my legs are good, my eyes aren’t great, but they’re okay…and I just take things…as they come…

P7 (PCA)

Participants also permitted themselves to say no to activities that were not enjoyable, or that primarily focused on abilities that were impacted by their diagnosis, rather than on their strengths.

*Subtheme 2:* acceptance as a process – ***‘it is what it is’***

Participants struggled with initially trying to hold tightly to previous aspects of themselves that were lost or changed by dementia, trying to *‘fight against’* (P6 [PCA]) the symptoms they were experiencing, before navigating a process of acknowledging, adjusting, and, in some cases, accepting these ongoing changes. Participants went through a *‘mourning period of coming to terms with it’* (P12 [PCA]), and accepting their own limitations, and the limitations of others. Some participants had been able to try to make adjustments where feasible, while also trying to accept aspects of their diagnosis that were outside of their control.

…I surprised myself that I was able to embrace that new image or landscape in front of me….

P3 (PPA)

There were specific losses that participants did not yet feel able to accept, particularly losses related to an important aspect of their identity.

…I’m a middle-class, educated woman, and I know not being able to read…that’s a hard thing…

*P5 (PCA)*

*Subtheme 3:* asking for and accepting support – ***‘it’s a strength to say I need help’***

Participants felt grateful for support and shared that they could not have managed without this. ‘Helpful’ support included: being asked what help was needed, ‘*spontaneous help’* (P5 [PCA]) and being given more time to do activities. Several participants struggled between feeling grateful for support and finding it difficult to accept their own limitations. It was often a difficult process of ‘*letting somebody support me rather than feeling like I don’t deserve or want to deflect the attention from me…’* and *‘feeling a bit more comfortable with being the focus than I used to be…’* [P3 (PPA)].

Participants tried to find a balance between support and independence. ‘Unhelpful’ support included people talking over or for the person with dementia and having to overly rely on others. In some cases, family members took over specific roles and responsibilities in an attempt to help, which led to a tendency to *‘overdo it a little bit’* (P7 [PCA]) resulting in a loss of independence and agency. In other cases, family members tried to encourage participants to continue to do tasks that they felt that they could no longer do, like trying to *‘include you in conversations’* (P2 [PPA]), which then made participants feel uncomfortable. Some participants also felt frustrated that other people *‘were saying … ‘No, you can’t do this’… they weren’t giving you a chance to see if you could still do it…’* [P4 (PCA)], diminishing their sense of self-worth.

Support preferences differed between participants. For instance, while several participants expressed frustration at people speaking on their behalf, others preferred friends and family to speak for them or to ‘*try and put the word in’* (P6 [PCA]. Participants expressed the desire to not only be someone who needed to be helped, but to be able to help and support others as well.

***Theme 4:*** changes in relationships – **‘things aren’t what they were’**

Participants spoke about other people distancing themselves, but also acknowledged that the change in relationships was partly due to their reduced ability to connect with others for example *‘…because of []speech hesitancy…’* (P3 [PPA]). Several participants did not want to overly rely on people around them, or to become a ‘burden’ on family members—now or in the future, emphasising the importance of others having *‘a break’* (P13 [PCA]). Participants acknowledged that those close to them were also experiencing feelings of loss. While it was not within the scope of this study to explore the relational aspect of loss further, this would be an interesting avenue for future research.

All participants also touched on positive aspects of relationships and the support they had received from people around them.

*Subtheme 1:* awareness and understanding – ***‘it’s very difficult to get across to people how it’s affecting me’***

Several participants felt that specific friends and family members had some understanding of their sense of loss, particularly if they were very close or if they had their own experience of living with a diagnosis or significant loss. All participants had at least one supportive friend, family member or professional. Several participants also shared that they had a wide support network and that telling people about the diagnosis was ‘*one of the best things we did’* (P14 [PCA]). Friends and family members also shared comments like *‘you’re just like you’ve always been’* (P5 [PCA]), reminding participants of their sense of continuing selfhood.

While some people took the time to understand their experiences, others had stepped away from participants due to their diagnosis, and were perhaps not able or willing to understand their experience, with a sense that*….’there’s [P13], he can still talk, he can still walk, he can still have a laugh…and therefore he’s all right…’* (P13 [PCA]).

Participants were not always able to share the more difficult aspects of living with their diagnosis and felt that they needed to pretend that they were doing or feeling better than they actually were, despite ‘*in the back of my head I’m thinking there’s so much of a change in me…’* (P10 [PPA]). People also made assumptions, often without asking about or listening to the reality of participants’ experiences. This was particularly relevant given the atypical nature of their diagnoses and the comparison to those living with more typical forms of dementia, with a common assumption being that ‘*dementia equals Alzheimer’s’* (P9 [PPA]). Some participants felt that most people ‘*could not know’* about their experience of dementia ‘*unless they were having these experiences…’* (P7 [PCA]).

*Subtheme 2:* peer support – ***‘you realise there’s a whole lot of other people dealing with this’***

Most participants found it helpful to meet other people living with non-memory-led dementia and to share difficulties in a friendly group where ‘*there’s always a laugh in it’* (P7 [PCA]) and group members are *‘all diagnosed with something similar’* (P2 [PPA]). Several participants living with PCA found it particularly helpful to have a group just for people living with dementia and a separate space for carers. Several participants also made connections with specific members of the group who were a source of hope for ‘*how they actually deal and have dealt with their challenges’* (P12 [PCA]) and also connected and formed friendships with group members outside of the peer support setting.

Not all aspects of peer support were perceived as helpful. Several participants living with PCA shared difficult aspects of seeing the progression of symptoms in others, leading to concerns for their own future. Participants also emphasised that while other people living with the same diagnosis likely shared aspects of their experiences, they were cautious of not wanting to over-generalise, as *‘I don’t know what other people’s … experiences are …so I don’t want to say you know ‘you can have…a good life’. I don’t know if that’s true…’* (P7 [PCA]).

***Theme 5:*** what helps in navigating loss – **‘the compensations that take the void away’**

Participants shared a number of strategies that they found helpful in navigating their experiences of loss.

*Subtheme 1:* what matters most **– *‘carpe diem’***

There was a general sense of reframing what matters most while living with dementia and that participants ‘*haven’t sort of stopped things in … life’* (P8 [PCA]).

Several participants felt that they had lost their sense of purpose after their diagnosis and were in the process of trying to re-evaluate and reconnect with this. Being supported to take part in activities, such as hobbies, research, and social groups, gave participants a sense of *‘being a part of society and people’* (P7 [PCA]). Activities included cooking, crossword puzzles, knitting, embroidery, advocacy, spending time with pets, Wordle, reading, listening to music, singing, volunteering, exercise classes, walking, connecting with nature and going on holiday. Participants also shared the importance of educating themselves and others about their condition ‘*to acknowledge….the rare dementias are out there*’ (P12 [PCA]).

Several participants also spoke about the importance of living in the moment, focusing on what they enjoyed and appreciating things more.

*Subtheme 2:* humour as a coping strategy – ***‘it’s important to have a laugh’***

All participants used humour throughout the research conversations, whether through self-deprecation of personal abilities, in response to the research conversation process itself, or as a general coping strategy. Humour was also used by participants to provide reassurance to others that it was OK to laugh together.

…….I am grumpy….sometimes I know I’m grumpy (P14 and wife look at each other and laugh)…

P14 (PCA)

However, one participant with PPA also shared that their diagnosis had changed the way that they were able to share their sense of humour with others, given the impact on verbal communication abilities.

*Subtheme 3:* feeling grateful for what I have – ***‘I think I’ve just been very lucky’***

Most participants shared that although living with a diagnosis of dementia was incredibly challenging in many ways, there were also many aspects of their lives that they felt lucky in or grateful for. These aspects included being in good physical health, being included in important life events, and spending time with family, friends, and animals. Several participants felt that many people were *‘worse off’* (P2 [PPA]) than themselves. Participants particularly emphasised the role of others in their well-being, and that it must be difficult for others to manage if they did not have access to this support. Not all comparisons to others were helpful, however, particularly given that several participants were living with a young onset diagnosis, and some expressed the unlucky nature of this.

### Terminology

**Figure 2.**
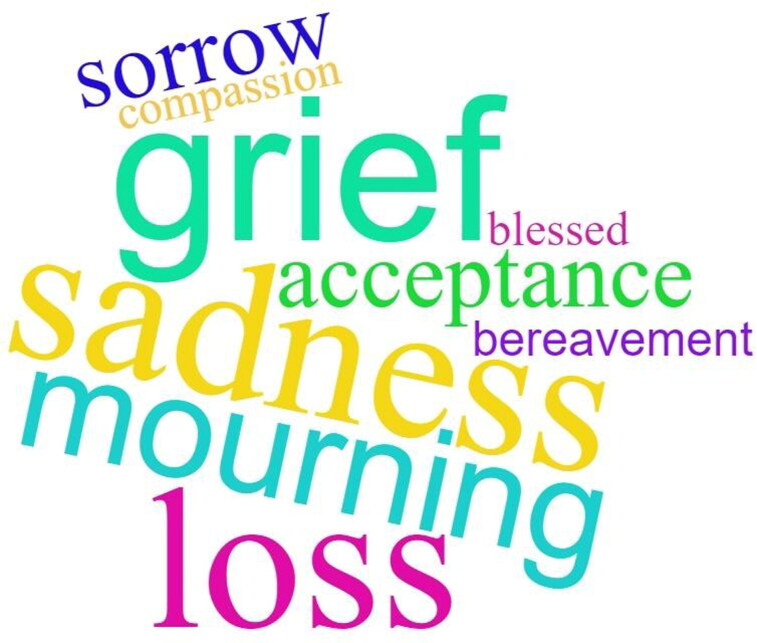
Grief terminology word cloud.

Participants were asked whether there was a particular word or phrase that reflected their experience of loss. The terms ‘grief’, ‘mourning’, ‘sorrow’ and ‘sadness’ were given as examples, with the option for participants to provide their own word or phrase to describe their experience. Responses are captured in the *Appendix* and visualised in Figure 2. The range of responses highlights the importance of individual preferences in relation to terminology when supporting people living with dementia in navigating loss.

## Discussion

This study explores the grief experience of people living with PCA or PPA, who shared losses related to specific activities, such as communication, driving and employment, changes in relationships and accepting support. Strategies used to navigate these losses included redefining what matters most, using humour and feeling grateful.

These findings connect to several grief-related theories which have been highlighted in previous research on grief in people living with dementia, including non-finite loss (Bruce & Schultz, 2001; Harris & Winokuer, 2019), chronic sorrow (Roos, 2002) and disenfranchised grief (Doka, 2010, 2019). The fluctuation between participants letting go of what they had lost, and holding on to the skills and abilities that remained, also closely connects to the Dual Process Model (Stroebe & Schut, 1999). This model has previously been applied to couples affected by dementia (Colquhoun et al., 2019; Merrick et al., 2016; Robinson et al., 2005), and this study expands this to include the intrapsychic process in people living with dementia. The interplay between holding on and letting go is complex, individual and nuanced, and highlights the importance of acknowledging, validating and understanding the experience of living with dementia as a whole, to provide both support for what is lost, and empowerment for what remains.

While there were many unique sources of loss, three aspects were common across participants: communication, employment and driving. Communication difficulties are a core feature of PPA (Marshall et al., 2018), a common symptom in the early stages of PCA (Schott et al., 2017; Yong et al., 2023) and an important aspect of fear of future progression in people living with Alzheimer’s disease (Cotrell & Hooker, 2005). Communication difficulties are a source of multifaceted grief in PPA, due to the ongoing impact on daily activities, roles and sense of self (Lo et al., 2022). Participants felt they had lost the ability to share important aspects of themselves with others, and this loss had impacted their relationships, leading to a sense of isolation.

Given that most participants in the present study were living with young-onset dementia, it was perhaps unsurprising that several participants viewed cessation of employment as another significant source of loss, which has previously been shown to be a vital source of social connection, sense of purpose, daily routines and identity for people living with young-onset dementia (Colquhoun et al., 2019; Smeets et al., 2024). Importantly, however, not all participants wanted to continue working, or found the loss of employment a source of grief. Driving cessation appears to be another common grief trigger point for many people living with different forms of dementia (Byszewski et al., 2013; Liddle et al., 2013; Sanford et al., 2019). This is particularly evident for those with a younger onset diagnosis (Scott et al., 2023), and relates to freedom, independence and identity.

Living with a dementia diagnosis impacts close relationships and wider social networks, due to difficulties with memory, communication, fear of becoming a burden and dementia-related stigma (Colquhoun et al., 2019; Doka, 2010; Jackman et al., 2024; Robinson et al., 2005). Participants shared several relationship-related losses, but also gains, particularly in strengthening connections within partnerships, focusing on what matters, and the value of peer support, which has also been emphasised in more typical forms of dementia (Colquhoun et al., 2019; Merrick et al., 2016; Pearson et al., 2022; Snyder & Drego, 2006).

Connecting with a sense of purpose, advocacy, maintaining independence, goal setting, companionship, having a positive outlook, accepting support, seeking help and helping others, using humour, and living in the moment have been similarly reflected in previous research specific to dementia (Colquhoun et al., 2019; Robinson et al., 2005; Snyder & Drego, 2006; Waddington et al., 2024) and in research on loss in older adults (Garrett, 1987).

Interpersonal gratitude, relating to support and relationships, feeling lucky and comparing oneself favourably to others have also previously been shown to be helpful coping strategies for people living with dementia (Pearson et al., 2022), with the current study widening these findings to include people living with young-onset dementia. The emphasis on the role of acceptance and re-evaluating what matters following a dementia diagnosis also indicates that Acceptance and Commitment Therapy (ACT) (Hayes & Pierson, 2005;Robinson & Moghaddam, 2022) may be a helpful component of a support group interventional framework, although this requires further exploration.

### Limitations

Limitations included that people living with forms of dementia other than PCA or PPA were not represented and may experience different symptoms, which may be primarily memory-led, and may lead to different grief-related experiences and coping strategies. Future research in this area could benefit from exploring the experiences of individuals living with other forms of dementia, including Alzheimer’s Disease, vascular dementia, lewy body dementia and FTD, to further understand the complexities of grief and loss in dementia. Research conversations were only conducted at one time-point, which does not reflect the progressive nature of dementia, or the changing nature of grief, loss and acceptance. Due to the nature of the study, only participants in the early stages were included and participants were recruited *via* a peer support group, meaning that they were already accessing a support service, and may be more open to discussions on loss compared with those who are currently not accessing support. The online component of the study may have excluded some participants from taking part, however this may have also increased accessibility for those who find travel difficult. Additionally, while the study participants were drawn from a typically under-represented research group, given they were living with non-memory-led, typically young-onset diagnoses, there was a limited range of participant ethnic, cultural and socio-economic backgrounds, which is important to address in future research.

### Future research

The current study underscores the significant impact of grief and loss in the experience of people living with early-stage PPA or PCA. Given the complex, in-depth and personal nature of the insights generously shared by participants, there is likely both a need and a benefit in accessing support that is tailored to these specific individual and shared experiences. Additionally, given the relatively preserved insight of people living in the early stages of these conditions, there is an indication that talking therapies may benefit and be appropriate for this group (Yong et al., 2023), although this requires further research. These findings, alongside existing grief-related intervention frameworks and the Medical Research Council guidelines (Skivington et al., 2021), will be used to develop, together with people living with dementia, a support group intervention to address the grief-related needs of people living with these diagnoses.

## Appendix 1

*Research Conversation Topic Guide*

***Question 1:*** *What term do you prefer to use when talking about your diagnosis?*

*Background*

*Sometimes people feel that they experience loss as part of living with (preferred terminology for diagnosis). There might be several different losses that you could experience. This can be very individual and unique to each person. I would like to ask you some questions about your own experiences. Please feel free to tell me as much or as little as you feel comfortable with.*

- *What are the most important losses you have experienced as a result of living with (diagnosis)?*
  - *Can you tell me about the impact of those losses on you?*
- *How do you feel these losses affect your ability to do the things you* ***need*** *to do?*
- *How do you feel these losses affect your ability to do the things you* ***want*** *to do?*
- *When are you most aware of these losses?*
- *Do you spend more time focusing on these losses*
  - *or*
  - *Do you spend more time focusing on other things?*
  - *Can you tell me more about what you spend most time focusing on?*
- *Do other people understand your losses associated to living with (diagnosis)?*

*Some people might be aware of the losses you experience and may be encouraging and supportive. Others might be unaware or don’t know what to say.*

- *What are some* ***helpful*** *ways that other people respond?*
- *What are some* ***unhelpful*** *ways that other people respond?*

*Some people living with (diagnosis) have support that they find helpful to manage with their losses. Some people also have support they find unhelpful when managing with their losses.*

- *What is* ***helpful*** *for you?*
- *What is* ***unhelpful*** *for you?*
- *Can you tell me about life before your diagnosis?*
- *Can you tell me about losses during that time*
- *You have told us about losses you have experienced as part of living with (diagnosis).*

*Some words people use to describe loss include:*

- *Grief*
- *Sorrow*
- *Sadness*
- *Mourning*

- *Do any of these words fit with your experience? Is there another term, word or phrase that fits better with your experience?*
- *Is there anything else you would like to add?*

## Appendix 2. Themes and illustrative quotes.`

**Table:**

*Theme 1:* what I have lost, am losing and will lose**: ‘I am not the old me’** ‘…. there was a point at which I thought….that really, kind of, I’ve gotta get used to it because I think this journey is going to involve quite a lot of recurrent losses. That’s …that’s quite a hard thing to realize…’ P5 (PCA)	
*Subtheme 1:* losses that came before my diagnosis – **‘*they live with me, even now’***	‘…when it comes to me…I don’t want it to be like it was with my mum…I want to be in a hospice…’ P5 (PCA) ‘…when you get to sort of your forties…not everybody, maybe, but…certainly me…you don’t take your body for granted…that’s the bit when you think right…I need to keep using it or losing it. I need to, you know, do a bit of weights, so I need to do a bit of cardio. Well, maybe more than just a bit to keep my body going (laughs), because you know this there’s bad stories about people…’ P10 (PPA) ‘….She was such a character and you know all these weird and wonderful sayings that she had. You know they live with me…()… So even now she still stays a big part of my life because of her humour and her wit …’ P12 (PCA)
*Subtheme 2:* what does my diagnosis mean? – ***‘it’s a terrible shock at the start’***	‘…So the funny thing is when you when I got cancer, it was ‘Oh, we’re sorry to hear that, you …can fight it. You can get through that.’ You say you’ve got dementia and they do a runner…’ P4 (PCA) ‘…being told you’ve got what I now call the ‘D Word’ in your forties, or fifties…or even sixties, you know. I just think people…don’t realise they can get that at that age. So it’s a double shock…’ P10 (PPA) ‘…it was only after diagnosis that you realized in pre-diagnosis, what you were losing and how important those things were. But in pre-diagnosis, you’re always trying to…find a reason or find logic as to why things are happening…uhm…and you just keep on going…’ P12 (PCA)
*Subtheme 3:* what does my future hold? **– *‘redefining who I was going to be’***	‘…I am concerned about me being non-verbal as this disease progresses further and the effects of dementia…’ P1 (PPA) – written response ‘…the first very strong feeling of loss of my future. So I’d got an image of myself going into retirement …I’d see myself as the old man with a shed …fixing things or making things but always being there as a person people could go to. So the first thing that hit me was that I’d lost all that. Hit me like a runaway train really.…I was inconsolable for I don’t know how long but I had to come to terms with the fact that I wouldn’t be able to be that person. So I had to start a process of redefining who I was going to be…’ P3 (PPA) ‘…I genuinely have no idea …how PCA works, I don’t know what’s going to happen next…’ P6 (PCA) ‘…I suppose, part of the thing about my whole life that I’ve had is always had a plan. I’ve always had a plan, you know, a plan for a career, and then a plan to get married, and a plan to have kids, and how many kids… I think, the plan I had already got has had to go, and that’s been the biggest shock for me…’ P10 (PPA)
*Theme 2:* shared and unique sense of loss – **‘you lose an essence of you’** ‘…I think, one of the…biggest loss was losing…my books…to actually hold a book instead of a kindle…it’s a very, very different thing. . .’ P6 (PCA)	
*Subtheme 1:* communication difficulties – ***‘I lost my sense of putting out who I was’***	‘…Used to be a fast talker…()…gossiping…now that’s all gone’ P1 (PPA) P2: ‘…you’ve got to think…before you speak…’ Researcher: ‘…OK…so that had an impact on your friendships…? P2: ‘…things aren’t what they were’ P2 (PPA) Researcher: ‘…what are the most important losses you have experienced as a result of living with PPA?’ P11: ‘…loss of speech…’ P11 (PPA)
*Subtheme 2:* loss of employment – ***‘I didn’t have a reason for being’***	‘…I only stopped work you know, it was a few months ago…but you’ve got all these things that you think you’re going to do and it your life just changes…’ P8 (PCA) ‘…I’d recognize that I’d…I’d started to lose words, and particularly names and…and that sort of thing. Yeah, I cut it, cut it short. ….I needed to…retire. I needed to give it up…and there wasn’t any other obvious way of getting back into work….’ P13 (PCA) ‘…really what I was recognizing was, was that after 50 years of working and developing and getting into construction and being…a…fairly serious part of…of the organizations I’ve been with it. Is it just gone…and that meant that I…I…I didn’t have a reason for being in some ways…’ P14 (PCA)
*Subtheme 3:* driving cessation **– *‘it’s a freedom which just disappears’***	‘…it’s a big loss as my husband doesn’t drive…()…I feel much cut off’ P2 (PPA) ‘…it’s… it’s the open air… the country….and you…it’s as if you have a care in the world…you’re just free…and then you lose all that…’ P4 (PCA) ‘…so it’s difficult to say how specifically, because… a whole lot of things automatically are suddenly wiped away….for instance…. .….I can’t drive the car…so it….which I’ve done for years…about a 1 million miles I think (laughs)….and so that’s an that’s an enormous difference, I understand why….but it is a freedom which …just …disappears…’ P14 (PCA)
*Theme 3:* balance between what is lost and what remains **– ‘I’ve lost a lot of things but I’ve also managed to hang on to lots of things’** ‘…loss is…a…good word … for different things, because you can put lots of different things onto that, you know, because …()…you have lost a lot of things…I’ve lost a lot of things, but I’ve also managed to hang onto quite a lot of things….and you know it’s… I do feel that it’s possibly a lot more difficult for other people and in that way you can also…whilst you can be lost with lots of things, you can also be blessed…’ *P8 (PCA))*	
*Subtheme 1:* challenging own abilities – ***‘don’t look at your mountain, climb it’***	Researcher: ‘…and doing Wordle, what does that bring for you?’ P2: ‘…I find it a challenge’ Researcher: ‘…and is it a positive challenge, so not feeling frustrating for you?’ P2: ‘No….No!…I usually get it….’ P2 (PPA) ‘…I’ve also in some ways got a sense if I don’t do something I’ve felt a bit apprehensive about before, I’ll never get round to it. So foreign travel has been something I’ve been afraid of because I’m afraid of flying. But I’m more of the mind-set I’m just going to do it anyway and enjoy the opportunity that it provides me. Whether I carry that through you’ll have to watch this space (smiles), but I’m feeling that whilst I’ve lost some things I want to do, I may gain some things because of my new outlook on the world…’ P3 (PPA) ‘… we all have black days…and you think yeah, I wish I could do something or whatever…but I much rather….get on with life, normally…’ P14 (PCA)
*Subtheme 2:* acceptance as a process – ***‘it is what it is’***	Researcher: ‘And are there any other words or phrases that maybe fit better with your experience?’ P1: ‘It is what it is…(laughs)’ P1 (PPA) ‘…it was making peace with the fact that I was going to have a shorter time and coming to terms with that realisation.…’ P3 (PPA) ‘…I don’t actually blame them, because I might have been the same way…[. .] ‘You fear the unknown’ and dementia is the unknown, so they stay clear of it…’ P4 (PCA)
*Subtheme 3:* asking for and accepting support – ***‘it’s a strength to say I need help’***	‘…That is it the difference between like being privileged that they care, and the other bit where it seems strange when you’re you know a 56-year-old man who then has got this young lady keeping an eye on me, you know, outside the toilet till I come out, so that we she can make sure that I make it safely back to the table…’ P8 (PCA) ‘…Sometimes people talk for me…which is really, really annoying. I hate that! I hate that, I don’t like being talked… you know, it’s not that kind of, ‘Does he take sugar?’ thing….But people think they’re helping by talking, and if I can’t find a word, people normally are quite patient for me to find an alternative, but I know it’s kind of a bit annoying if the conversations flowing but… people will….I don’t know sometimes I don’t mind a bit of help in those situations. Most people are pretty good…’ P9 (PPA) ‘…And now I do actually say to people, ‘Look, I am struggling with…with words. Now do you know you’re just going to have to forgive me. Give me a little bit of space but if I really can’t work out what it is please just fill in the blank’, you know because I don’t want people automatically jump into the conclusion that they need to do it straight away. So that’s a recent loss, and the one that’s really put me in a bad place, but I’m not too bad with it. But it was a shock’ P12 (PCA)
*Theme 4:* changes in relationships – **‘things aren’t what they were’** ‘…I think, ah, my husband…loss….I can’t find the words to say to him….’ P1 (PPA)	
*Subtheme 1:* awareness and understanding – ***‘it’s very difficult to get across to people how it’s affecting me’***	‘…I had a bad…experience with a social worker….()….the …first thing he says to me was…’You’ve got dementia…you can’t be on your own with dementia, you need to be in a care home’….’ P4 (PCA) ‘. .somebody with dementia’s got two faces….so you can come across that …nothing’s wrong….but…underneath it is because you’re you’ve lost a lot of things. There’s a lot of things you can’t do…’ P4 (PCA) ‘…I think some people probably understand quite a bit of it. My husband, I think, does understand definitely more than 50%. I don’t know whether all my kids do, because I don’t want them. I didn’t want to be that burden to them. I think my daughter, who’s the oldest kid I’ve got. I’ve had a bit more of an honest chat with her so, but I feel for her a bit… we’re very open as a family, so does…. know, I think some of it. But yeah, I think some people know the impact on me to some extent. I don’t think…if I’m honest, I don’t think anybody apart from maybe you (researcher), know the full impact…’ P10 (PPA) ‘…I find that interesting that many people say, you know some people do understand, some people don’t want to understand, and some people don’t get it and they…they have the approach ‘well, you’re just having a bad day’ and to me, if somebody says that to me, I actually will move on to a different conversation …()…I don’t flog my PCA…to anybody. There’s no point…but at times it is quite nice to kind of say, oh, this has happened, or that’s happened and some people take an interest and some people, don’t, and for most part people say, ‘Oh, come on, P12, it’s not that difficult, what you’re talking about. Go and butter some bread’. At that point I mentally turn off…’ P12 (PCA)
*Subtheme 2:* peer support – ***‘you realise there’s a whole lot of other people dealing with this’***	‘…at the very start I…I used to sit back and just observe and listen….And then I used to watch people on how they reacted to questions, and…then I used to think, ‘Well, if he can do it, I could do that….uh… there is no difference, he he’s got dementia, I’ve got dementia…if he can get up there and talk why can’t I?…’ P4 (PCA) ‘…nobody knows if my eyesight is going to get worse and going to the PCA meeting it appears that some of them do get worse. Some people’s eyes get worse, which is…a bit concerned, because I really don’t want to lose what I’ve got left…()… So you know it’s, but it’s, and that’s quite frightening, actually going to that PCA group, because I can see what might be happening to me in the future because some people are ahead of me, if you like.’ P6 (PCA)
*Theme 5:* what helps in navigating loss – **‘the compensations that take the void away’** ‘….But my big plan now is ‘carpe diem’, it’s ‘seize the day’, enjoy what you do…()…so I’m doing a bit more of that, you know we’re doing more holidays…I’ve always loved music, and one thing that I’ve noticed that I’m loving still is music…so we’re going to quite a few gigs. We’ve done quite a few already this year, and we’re gonna do more, so I don’t know…I’m trying this one aspect to me, which is a bit more you know. Enjoy what you enjoy and seize the day. So there’s a bit more of that positive side of me…’ P10 (PPA)	
*Subtheme 1:* what matters most ***– ‘carpe diem’***	‘…I…look at the woods, and the robin comes to meet me….I enjoy the plants and animals….’ P2 (PPA) ‘But I’ve also, at one point, definitely decided that the things I can, do, I’m going to keep doing for as long as I can …you know and I’m not going to give anything up until I have to, unless I want to. I’m allowed to do it, if I want to (laughs)’ P5 (PCA) ‘…I can make this go on for a while. The moment you stop, everything, I mean…you’re sunk then aren’t you? You’ve gotta keep something going…’ P9 (PPA) ‘…I don’t remember …feeling that bad because from the day we started… was the… idea was to do the to do what we could, when we could…and get and have the best life we could do. And that’s… something which, if I may (turns to wife and holds her hand) say to… (my wife) that she has been my rock…’ P14 (PCA)
*Subtheme 2:* humour as a coping strategy – ***‘it’s important to have a laugh’***	‘It’s like one in ten people really sees the person you are…and that might be via your sense of humour, or your view on the world…you’ve been noticed by somebody, you’ve been valued by somebody. They might have said “I really like your sense of humour, I like where you’re coming from”…’ P3 (PPA) ‘….I think some people have said they think I’m a bit quieter than I used to be. I think I used to be one of the big chatters (laughs)…’ P10 (PPA) ‘…I’m happy to keep on going. I’m in my stride (laughs)…’ P12 (PCA)
*Subtheme 3:* feeling grateful for what I have –***’I think I’ve just been very lucky’***	Researcher: …’and what do you find helpful about reading that blog?’ P2**…** ‘Somebody is much worse than I am (laughs)… ‘ P2 (PPA) ‘…I have so much support like It’s…it’s amazing. I feel really lucky…I don’t know what, whether other people have all…have that same support and like loving care…uhh…I’m very lucky I think…’ P7 (PCA) ‘…I did step back and think, P10 don’t beat yourself up, there’s obviously people with way worse scenarios than where I am right now, I think you know. So I do I do focus sometimes…. .Well, probably more than doing all the scary stuff focus on what you’ve got, not what you’ve lost. .’ P10 (PPA) ‘…I feel bad saying this, but you know, if I’d got to eighty and only got Alzheimer’s, well ‘only got’ Alzheimer’s, Alzheimer’s is bad too…I could probably now get my head around that…’ P10 (PPA) ‘…I don’t do it very often and if I do it, it’s usually me thinking…uhm… don’t be so stupid (laughs)…you know, you’ve got a lovely family. You’ve got lots of people. You’ve got lots of friends. Just get on with it…’ P14 (PCA)

## Appendix 3

Terminology used by participants to describe their experience of loss related to living with a diagnosis of dementia.

**Table:**

P1 – grief, mourning	P1: ‘Grief…. .Mourning’ Researcher: ‘And are there any other words or phrases that maybe fit better with your experience?’ P1: ‘It is what it is (laughs)’
P2 – grief, sorrow and sadness	Researcher: (reading chat) ‘thank you…so you’ve said grief, sorrow and sadness feel like they fit most with your experience?’ P2: ‘Yeah’ Researcher: ‘And is there one of those words that fits the most for you, or do they all…?’ P2: ‘Sorrow’ Researcher: (repeats) ‘Sorrow.’ P2: ‘ That sums it up’
P3 – mourning, grief, sorrow, sadness, acceptance	P3: ‘…I’m mourning the loss of my future, length of life…Grief I think suggests to me a more short-term, ‘hit me’…grief…realising what I’m fighting. Mourning is almost like “I’m at the beginning of acceptance of the new situation”…I suppose sorrow is kind of wrapped…with mourning to me…sadness….they’re kind of all part of the transition from immediate impact to acceptance of living with it. So it’s almost as if they’re different points of the graph as you come down to new normality going forwards. So I would say they all have a relevance to me how I feel going through the stages of where I am today from my initial deterioration…’
P4 – loss	P4: ‘…like the driving, I wouldn’t call that grief. I would say it’s a loss…more than…I would definitely say that when all the family passed away…’
P5 – grief	P5: ‘…Yeah, I think it was grief…()…That helped me to label it as grief…’
P6 – sadness	P6: ‘I think it’s three of them I find very emotive. So I would say, sadness….Yeah….personally, yeah…’
P7 – sadness, acceptance, compassion	P7: ‘I think I would say sadness?’ Researcher (repeating): ‘Sadness’ P7: ‘Yeah, sometimes….because everything changes, and it is sad, it’s very sad…but I would also use the word acceptance….it’s really helpful, and compassion…’
P8 – loss, blessed	P8: ‘…loss is… is…is a… a… good…good word of…of, you know from for different things, because you can put lots of different things onto that, you know, because you…()…you have lost a lot of things…I’ve lost a lot of things, but I’ve also managed to hang onto quite a lot of things….and you know it’s… I do feel that it’s possibly a lot more difficult for other people and in that way you can also…whilst you can be lost with lots of things, you can also be blessed…’
P9 – loss	P9: ‘…Yeah. That’s probably more for the carer or the partner of that person.…I don’t feel any of those. I don’t feel sorrow…loss is just the word I think (researcher), I don’t think it should be any bigger, or grander or anything other than loss….you just lose stuff….()….I mean, you know, other people, what I was…coming back to your question, I think, you know, sorrow, grief, those are things that people around you who love you would say, it’s not what you experience…’
P10 – grief, grieving	P10: ‘I’m grieving in lots of aspects. I’m grieving because of the shock, because of the word terminal and grieving because I feel like I’m not the old me in lots of aspects…’
P11 – loss	Researcher: ‘Is there another word or phrase that fits for you and your experience?’ P11: PA reading phone: ‘Loss’
P12 – bereaved, mourned, sad, loss	P12: ‘Yeah it’s strange and it isn’t something I’ve thought about when I’ve used the terminology, it must be how I’m feeling inside just relates to a particular word…()…I’ve mourned the loss of my business. I felt bereaved when I had to give my business up when I had made that decision. That was a terrible decision to have to make…()… I don’t think I’ve ever really used the word grief and I don’t know what the I can’t remember the other words you said, but I don’t…’ Researcher: ‘sorrow was one…?’ P12: ‘I haven’t used the word sorrow…I have on occasion used the word sad…for me it’s bereavement. It’s loss. It’s learning how to overcome that you know mourning something that you’ve lost….and when I use that, I’ve used it in the context of it makes me sad of what I’ve lost but I have to keep going so sad is not…()…I would use loss, bereavement, mourning…’ ∼ P12: ‘I’ve lost the ability to put my clothes on without…knowing what my outfits are, if you know what I mean? I wouldn’t say, “Oh, I’ve grieved over that particular part in my life”, because I think that’s …that’s…it’s too… it doesn’t…it’s like over-egging the pudding in my head, because I don’t feel that strongly about it because of being able to find a solution, therefore I don’t need to use such a powerful word as grieved.’ ∼ P12: ‘The bereavement came in when I had to get my business up and the rest, the…rest are losses. If you can see where I’m differentiating there’ ∼ P12: ‘…I would class it as a bereave-loss…I will put it in both categories. That’s how I would class that, because that’s going to change my life. It’s all life-changing but some of it’s manageable, uhm…something like that in my opinion as that gets worse it’s unmanageable by the person that it’s happening to…’
P13: loss	P13: ‘…well of the losses that I’ve I experienced going through life…they have different…meanings sometimes….When I got PCA and…learned what was going on….I was angry more than… anything else…frustrated for… not understanding what… it was and frustrated, that …nobody could seem to be able to help….it…covered the feeling of loss from working…you know…the process of trying to get…the diagnosis and …get some help to that diagnosis…then the realization that that stage was…very much straightforward loss and there wasn’t any …other word for it…’
P14 – grief	P14: ‘…Uhm…that’s difficult…because it’s now a year ago….uhm…the…(pauses)…not, I wouldn’t use….yeah there were…there were days where I might think…think that grief might have been a….but it … would be. It would be a… single instance rather than an over…an overall…’
